# Angiographic severity of coronary artery disease and the influence of major cardiovascular risk factors

**DOI:** 10.4314/gmj.v57i4.2

**Published:** 2023-12

**Authors:** Nehemiah J Dung, Mark M Tettey, Martin Tamatey, Lawrence A Sereboe, Alfred Doku, Martin Adu-Adadey, Francis Agyekum

**Affiliations:** 1 National Cardiothoracic Centre, Korle Bu Teaching Hospital, Accra, Ghana; 2 Cardiothoracic Surgery Division, Surgery Department, Jos University Teaching Hospital, Jos, Nigeria

**Keywords:** coronary artery disease, cardiovascular risk factors, coronary angiography, SYNTAX score, influence

## Abstract

**Objective:**

To determine the angiographic severity of coronary artery disease (CAD) and assess the influence of major cardiovascular risk factors (CVRF)

**Study design:**

a cross-sectional, hospital-based study.

**Setting:**

the catheterisation laboratory of the National Cardiothoracic Centre, Accra, Ghana.

**Participants:**

for 12 months, consecutive patients admitted for coronary angiography were assessed for the presence of CVRFs. Those with significant CAD after angiography were recruited into the study.

**Intervention:**

The patient's angiograms were analysed, and the CAD severity was obtained using the SYNTAX scoring criteria.

**Main outcome measure:**

The lesion overall severity (SYNTAX) score and the relationship with CVRFs present

**Results:**

out of the 169 patients that had coronary angiography, 78 had significant CAD. The mean SYNTAX score was 20.18 (SD= 10.68), with a significantly higher value in dyslipidaemic patients (p < 0.001). Pearson's correlation between the score and BMI was weak (r= 0.256, p= 0.034). The occurrence of high SYNTAX score lesions in about 18% of the population was significantly associated with hypertension (OR= 1.304, 95% CI [1.13-1.50]; p= 0.017) dyslipidaemia (OR= 5.636, 95% CI [1.17-27.23]; p= 0.019), and obesity (OR= 3.960, 95% CI [1.18-13.34]; p= 0.021). However, after adjusting for confounding factors, only dyslipidaemia significantly influenced its occurrence (aOR= 5.256, 95% CI [1.03-26.96]; p= 0.047).

**Conclusion:**

Even though the most severe form of CAD was found in about one-fifth of the study population, its occurrence was strongly influenced by the presence of dyslipidaemia.

**Funding:**

None

## Introduction

Coronary artery disease (CAD) is the clinical manifestation of coronary artery insufficiency due to complex and chronic coronary artery narrowing caused by atherosclerosis (in 95%).^1^ As a growing global epidemic, CAD is the single most common cause of death in developed nations and a leading cause of disease burden in developing nations, with a projected incidence doubling by 2030.^2^,^3^ The rise in low-income countries has been attributed to the rising incidence of CVRFs resulting from the ongoing epidemiological transition.^4^

Although invasive and expensive, coronary angiography remains the gold standard investigation for CAD diagnosis. It enables the assessment of the lesion's adverse characteristics used in SYNTAX (synergy between percutaneous coronary intervention with taxus and cardiac surgery) score calculation.

The clinical state and the severity (SYNTAX) score of the lesions influence the choice of therapy.^5^,^6^

The Framingham heart study has established the role of major CVRFs (such as age, male gender, genetics/ positive family history, cigarette smoking, dyslipidaemia, hypertension, and diabetes) in CAD pathogenesis.^7^ These risk factors act as initiators and promoters of disease progression from an indolent initial atherosclerotic lesion to a complex and clinically significant form.^7^,^8^ However, despite the variable influence of CVRFs on the disease severity, studies have shown that identifying and controlling the dominant risk factor(s) slow(s) or stop(s) the progression of CAD to more complex forms.^9^ Whereas some investigators have reported hypertension as the most common CVRF of CAD in sub-Saharan Africa (including Ghana), there was no report of its influence on the disease severity.^3^,^10^ Also unknown is/ are the dominant CVRF(s) in the subregion, which, if adequately controlled, may halt or slow the development of the more complex lesions that require coronary artery bypass graft surgery.

Thus, this study sought to determine the angiographic severity of CAD using the SYNTAX I scoring system and to assess the influence of CVRFs on the disease's severity at the National Cardiothoracic Centre, Accra, Ghana.

## Methods

### Study design, setting and population

The study was a cross-sectional observational study on consecutive patients with positive coronary angiography diagnosed at the catheterisation laboratory of the National Cardiothoracic Centre (NCTC), Korle Bu Teaching Hospital (KBTH), Accra, Ghana. It was conducted over 12 months (between September 2019 and November 2020, excluding the three months of COVID-19 lockdown). Patients with significant or positive coronary angiography were included in the study. Significant or positive coronary angiography was defined as the presence of at least one epicardial artery measuring at least 1.5 mm in diameter (at least a first-order branch of the major coronary branches), with at least 50% luminal stenosis (2018 ESC/EACTS guideline).^11^ Those with positive angiography after a previous coronary revascularisation procedure were excluded.

### Sample size calculation and data collection

Due to the absence of data on significant CAD in this subregion, the sample size calculation was done using a point prevalence of 59.7% (a proportion of significant CAD from previous coronary angiograms conducted at the centre). After correcting for a finite population, an estimated minimum sample size of 73 was obtained.^12^

All participants (or their guardians) were issued informed consent forms, and the study's purpose, procedure, benefits, and risks were explained to them. They were also assured of the confidentiality, anonymity and protection of vital information. Those that consented were assessed for the presence of major CVRFs using the most recent diagnostic criteria as follows: diabetes mellitus defined as either fasting blood glucose ≥7 mmol/L measured at 2 consecutive periods, HbAlc ≥ 6.5%, or if the subject had been on medication for blood glucose control; hypertension as systolic blood pressure ≥140 mmHg with or without a diastolic blood pressure ≥90 mmHg, or when already on antihypertensive medication; dyslipidaemia defined as either a fasting lipid profile showing at least a total serum cholesterol >5 mmol/L, LDL-C >3.2 mmol/L, HDL-C< 1.3 mmol/L, or triglycerides >1.7 mmol/L, or when already on lipid-lowering drugs; and obesity as a BMI ≥30 Kg/m^2^ or a waist-hip ratio >0.90 in males and >0.85 in females. 14,15,16,17 Others include cigarette smoking, considered significant in current or previous cigarette smokers of at least a stick per day, or those with substantial exposure to second-hand tobacco smoke (we thought only spouses or roommates were exposed in most parts of the day or night); and positive family history in those with a history of atherosclerotic cardiovascular disease or sudden death in a first-degree relative before age 55 in males or 60 in females.^9^,^18^

**Figure 1:**
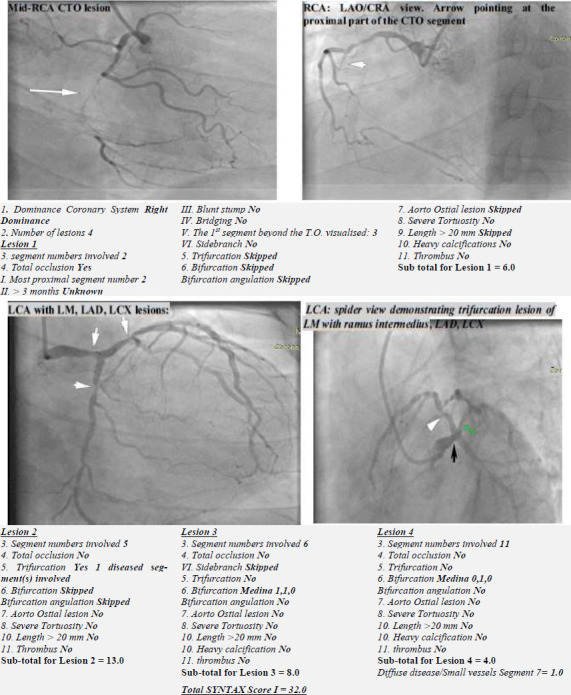
Coronary angiogram of a 50-year-old patient in the study, showing the SYNTAX score calculation LAO: Left anterior oblique, CRA: cranial, LCA: left coronary artery, LM: left main, LAD: left anterior descending, LCX: left circumflex

At least two cardiologists performed the coronary angiography through the radial or femoral artery approach using a tiger catheter or, occasionally, the Judkins catheters. Images were obtained using Siemens Artis Zee with Pure® catheterisation/ cardiac angiography system. The degree of luminal stenosis in each coronary artery segment was assessed by digital quantification and visual assessment of the stenotic segment compared to the nearest normal reference segment. Those with positive findings were enrolled on the study, while the SYNTAX score calculator was used to obtain each patient's overall SYNTAX I score, as demonstrated in Figure 1).^13^ The overall SYNTAX I scores were categorised into low, intermediate, and high-risk lesions for percutaneous coronary intervention (PCI), for scores ≤ 22, 23 to 32, and >32, respectively.

### Statistical analysis

Data collated was entered into a Microsoft Excel 2016 spreadsheet and analysed using the statistical package for social sciences (SPSS) software version 25 (IBM Corp. Released 2017. IBM SPSS Statistics for Windows, version 25.0. Armonk, NV: IBM Corp). Quantitative variables with a normal distribution, such as age, BMI and SYNTAX score, were expressed as mean and standard deviation. In contrast, qualitative variables such as CVRFs and SYNTAX score categories were expressed as frequencies and percentages. The results were presented as tables and charts. Pearson correlation analysis was conducted between the SYNTAX scores and the age and BMI, while an independent t-test was used to compare the mean SYNTAX scores among the categorical independent variables such as sex (male and female). A chi-square test was used to establish the significant associations between CVRFs and high-score lesions, while binary logistic regression analysis was used to obtain the adjusted odds ratio. A statistically significant level was set as p < 0.05.

### Ethical consideration

Ethical clearance was obtained from the Ethical and Protocol Review Committee (EPRC), College of Health Sciences, University of Ghana (Protocol identification number: CHS-Et/ M.10-P5.6/2018-2019).

Clearance was also obtained from the Head of the National Cardiothoracic Centre, Korle Bu Teaching Hospital, where the study was conducted.

## Results

One hundred and sixty-nine patients had coronary angiography between September 2019 and November 2020. Of these, 84 were significant, and 6 were excluded due to previous revascularisation procedure. Thus 78 patients met the selection criteria.

The mean age of the population was 60.53 (SD= 9.51) years (ranging between 43 to 84 years), with majority in the seventh (35.9%) and sixth (33.3%) decades. About three-quarters of the population were male, but the age difference between the female and the male population, was not statistically significant (p=0.105). The most common CVRF was systemic hypertension (76.9%), followed by dyslipidaemia, obesity, and diabetes. Most patients (44.9%) had clustering of at least three modifiable CVRFs (Table 1). The overall mean SYNTAX score was 20.18 (SD=10.69), ranging from 2 to 49. The mean was significantly higher in dyslipidaemic patients (p < 0.001), but lower in cigarette smokers (p=0.037), as shown in Table 2.

**Table 1:** Sociodemographic characteristics and risk factors of coronary artery disease

Variables	Mean (SD)/ n %	Test	p-value
**Continuous variables**			
**Age (years)**	60 (S9.50)		
**Female age**	63.50 (9.95)	1.641^t^	0.105
**Male age**	59.50 (9.21)		
**BMI (kg/m** ^**2**^ **)**	28.67 (6.04)		
**Female BMI**	30.26 (5.03)	1.377^t^	0.902
**Male BMI**	28.12 (6.26)		
**Categoric variables**			
**Age group in years**			
**< 50**	11 (14.1%)		
**50-59**	26 (33.3%)		
**60-69**	28 (35.9%)		
**70-79**	9 (11.5%)		
**≥ 80**	4 (5.1%)		
**Female**	20 (25.6%)		
**Male**	58 (74.4%)		
**Hypertension**	60 (76.9%)		
**Diabetes mellitus**	27 (34.6%)		
**Dyslipidaemia**	45 (57.7%)		
**Obesity**	29 (37.2%)		
**Cigarette smoking**	20 (25%)		
**Positive family history**	3 (3.8%)		
**1 MRF**	18 (23.1%)		
**2 MRFs**	25 (32.1%)		
**≥ 3MRFs**	35 (44.9%)		

*NOTE: SD: standard deviation, MRF: modifiable risk factor*

t *denotes independent samples t-test, p* < *0.05 is significant*

**Table 2:** Mean SYNTAX score difference among CVRF categories

Variable		Mean SYNTAX score (SD)	t	p-value
**Overall SYNTAX score**		20.18 (10.68)		
**Gender**	Female	19.92 (11.00)	−0.956	0.719
	Male	20.92 (9.95)		
**Diabetes**	Yes	17.26 (8.30)	−1.780	0.079
	No	21.72 (11.50)		
**Hypertension**	Yes	20.99 (11.00)	1.229	0.223
	No	17.47 (9.31)		
**Dyslipidaemia**	Yes	23.84 (10.68)	3.838	< **0.001**
	No	15.18 (8.56)		
**Smoking**	Yes	15.90 ( 8.53)	−2.123	**0.037**
	No	21.65 (11.02)		
**Obesity**	Yes	22.46 (11.38)	1.463	0.148
	No	18.83 (10.13)		
**Family history**	Yes	19.17 (4.65)	− 0.166	0.869
	No	20.22 (10.87)		

*NOTE: BMI: body mass index, SD: Standard deviation; t: independent samples t-test, p* < *0.05 is statistically significant*.

**Figure 2:**
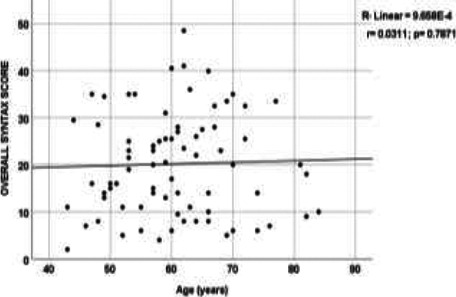
The correlation between age and the SYNTAX score

**Figure 3:**
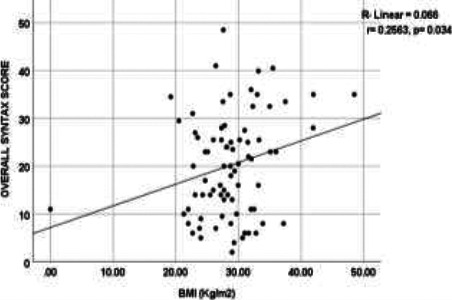
The correlation between the BMI and the SYNTAX score

As shown in Figure 2 and Figure 3, a bivariate correlation analysis between the SYNTAX scores and BMI showed a significant but weak positive correlation (r=0.256, p=0.036). In contrast, the difference between the SYNTAX scores and age was not significant (r=0.0311, p= 0.7871).

After categorising the lesions based on their SYNTAX scores, low-score lesions (≤ 22) were predominant (55%), followed by intermediate scores (23-32) and high-score lesions (> 32) in 27% and 18%, respectively. A chi-square test between the high SYNTAX score lesions and the CVRFs, showed a significant association with a 1.3, 5.6 and 4.0-odds in hypertensive (OR= 1.304, 95% CI [1.13-1.50]; p= 0.017), dyslipidaemic (OR= 5.636, 95% CI [1.17-27.23]; p= 0.019) and obese (OR= 3.960, 95% CI [1.18-13.34]; p= 0.021) patients, respectively. Similarly, the association was significant in the presence of at least 3 modifiable CVRFs. However, the wide confidence intervals suggest that the result should be interpreted cautiously **(Error! Reference source not found.)**.

After controlling for other risk factors using the logistic regression model **(Error! Reference source not found.)**, only dyslipidaemia had a statistically significant independent influence, with a 5.3-odds, on the development of a high SYNTAX score lesion (aOR= 5.256, 95% CI [1.03-26.96]; p= 0.047).

## Discussion

The role of cardiovascular risk factors in the initiation of CAD is well established, but their role in the progression to a high-complexity lesion warranting more invasive intervention varies.^7^,^19^ Similar to the report in previous studies,^5^,^20^ significant CAD in our study was most common between the sixth and seventh decades, with a mean population age of 60.53 years (SD= 9.51). Although several studies agreed with our finding of a 1:3 female-to-male ratio,^20^,^21^ a higher male proportion (1:1.5) was reported in a similar study by El-Kersh et al. in Menoufia, Egypt.^22^ Also, this study agrees with the report of hypertension as the most common CVRF in significant CAD in Ghana by Ofori-Asenso 3

Concerning the severity/ complexity of CAD in this study, even though the mean SYNTAX score of 20.18 (SD= 10.69) was comparable with the 19.1 (SD= 11.4) reported by Tanaka et al.,^21^ it was higher than the 15.0 reported in a similar study by El Kersh et al.^22^ The SYNTAX trial of 2005 classified the complexity of CAD based on the risk of major cardiovascular and cerebrovascular events (MACCE) following PCI.^23^ In our study, the low-score lesions (≤ 22) were the most common (55%), followed by intermediate-score (23-32) and high-score (> 32) lesions in 27% and 18% of the study population, respectively. Thus, regarding the relationship between the severity/ complexity of CAD and the various CVRFs in this study, a significant but weak correlation was established between the SYNTAX score and BMI (r=0.256, p= 0.036). In contrast, the correlation with age was not statistically significant (r=0.031, p= 0.7871). However, for unclear reasons, our finding contradicts that reported by El-Kersh et al., where only age had a strong positive correlation with the SYNTAX score (r=0.639, p < 0.001).^22^ Also, while many authors 22,24 agreed with our finding of a slightly higher but non-significant SYNTAX score in the male population (20.92 vs 19.92), Tanaka et al.^7^ reported a significantly higher male score. The likely reason for the non-significant difference in our study could be attributed to the older mean age of the male population (M= 59.5, SD=9.21), closing in on the female's mean age of 63.5 years (SD= 9.95). At such age, the severity of CAD in women rises towards that of the male population due to the post-menopausal loss of the protective, anti-sclerotic role of oestrogen.^21^,^25^ Furthermore, the significantly higher mean SYNTAX score in dyslipidaemic patients (p < 0.001) with about 5-fold adjusted risk of the high-score lesion (p=0.047) in this study agrees with those reported in other studies.^24^,^26^ Even though the wide confidence interval could be attributed to the small sample size, the significant relationship could still be related to the central role of dyslipidaemia in the pathogenesis of CAD,^7^ as well as the delayed diagnosis and institution of lipid-lowering medication (reported to be a common problem in this sub-region).^27^

In contrast, despite the significant role of diabetes in CAD pathogenesis, it was not associated with a significant occurrence of severe CAD (p=0.068). While other investigators have reported similar findings,^18^,^28^ Tanaka, El-Kersh and Bhattacharyya, reported contrary reports.^21^,^22^,^29^ The finding in our study could be related to the lower atheromatous burden associated with early medical treatment of diabetes.^21^

This is possible with the report of a significant level of oral antidiabetic drug adherence in Ghana (Bruce et al.).^30^ Similarly, despite the significant association between obesity and high-score lesions in this study (OR=3.96, 95% CI [1.18-13.34]; p= 0.021), the influence was lost after adjusting for confounding factors by logistic regression analysis (aOR= 0.339, 95% CI [0.09-1.24]; p= 0.102). This finding agrees with those reported by several investigators.^31^,^32^ Also, despite being the most common modifiable CVRF in our study and its significant association with high-score lesions (p=0.017), hypertension was not a significant independent predictor of high-severity lesions (p=0.998). Our finding agrees with those reported in other studies.^22^,^29^

Similarly, in agreement with several studies,^21^,^24^ cigarette smoking had no significant influence on the occurrence of severe CAD (p=0.072). However, contrary to El-Kersh et al.'s finding of a significantly high mean SYNTAX score among cigarette smokers,^22^ our study found a significantly lower mean SYNTAX score in cigarette smokers. While the role of cigarette smoking in the pathogenesis of CAD is well established,^7^ this finding could be attributed to the coexistence of multiple CVRFs [n=60, (77%)] in the study population. These other CVRFs in non-smokers [n=70, (75%)] could have contributed to the raised mean SYNTAX scores. Furthermore, the association between positive family history and CAD severity was insignificant (p=0.548). While our finding agrees with previous studies.^29^,^31^ the result could have been influenced by the few numbers of patients [n= 3 (4%)] that admitted to having a positive family history. Finally, in agreement with the report by Supariwala et al.,^33^ clustering of at least 3 modifiable CVRFs was significantly associated with high SYNTAX score lesion (p=0.036). This could be possible as multiple CVRFs are known to act in concert to increase the atheromatous burden by synergising each other's effect.^33^

Even though dyslipidaemia was significantly associated with the most severe forms of the disease, the study did not quantify the contribution of each component of the lipid profile or analyse the impact of lipid lowering drugs on the disease. Thus, further study is needed to identify the specific lipid responsible for the disease progression and the influence of statins. Secondly, the study population was small because it was a single-centre, hospital-based study over 12 months. These contributed to the wide confidence intervals in the risk assessments, making the interpretation of the findings uncertain. Therefore, we recommend a prolonged and multi-centre study involving a larger sample size from a broader spectrum of patients to verify the findings of this study.

We also recommend that intensified surveillance of dyslipidaemia be encouraged through early screening and aggressive lipid-lowering measures to slow the disease progression in the population to the high score category.

## Conclusion

In this study, only a fifth of the population had a high SYNTAX (severity) score lesions that often require surgical intervention. Despite the significant association between hypertension, dyslipidaemia and obesity with high score (complexity) lesions, only dyslipidaemia had a significant independent influence on the occurrence of the most complex/ severe form of the disease.

## Figures and Tables

**Table 3:** Factors associated with high SYNTAX score lesions

Variable	Odd's ratio	95% CL	P-value
**Age (>65 years)**	1.917	0.58-6.30	0.220^f^
**Male gender**	0.341	0.16-1.90	0.262^f^
**Diabetes**	0.260	0.54-1.26	0.068^f^
**Hypertension**	1.304	1.13-1.50	0.017^f^
**Dyslipidaemia**	5.636	1.17-27.23	0.019*
**Smoking**	0.182	0.02-1.49	0.072^f^
**Obesity**	3.960	1.18-13.34	0.021*
**Family History**	0.953	0.90-1.01	0.548^f^
**2 Modified CVRFs**	0.217	0.57-38.76	0.108^f^
**≥ 3 Modified CVRFs**	3.650	1.03-12.91	0.036*

*CVRF= = cardiovascular risk factors*

* *chi-square test*

f *Fisher's exact test; p*> *0.05 is significant*

**Table 4:** The independent risk factor(s) of high SYNTAX score lesion

Variable	aOR	95% C.I	p-value*
**Hypertension**	0.000	0.00-1.00	0.998
**Dyslipidaemia**	5.256	1.03-26.96	0.047
**Obesity**	0.339	0.09-1.24	0.102

* *Binary logistic regression analysis; aOR: adjusted odds ratio, CI: confidence interval, p* < *0.05*.

## References

[1] Sayols-Baixeras S, Lluís-Ganella C, Lucas G, Elosua R (2014). Pathogenesis of coronary artery disease: focus on genetic risk factors and identification of genetic variants. Appl Clin Genet.

[2] Gaziano TA, Bitton A, Anand S, Abrahams-Gessel S, Murphy A (2010). Growing epidemic of coronary heart disease in low-and middle-income countries. Curr Probl Cardiol.

[3] Ofori-Asenso R, Garcia D (2016). Cardiovascular diseases in Ghana within the context of globalization. Cardiovasc Diagn Ther.

[4] Dalen JE, Alpert JS, Goldberg RJ, Weinstein RS (2014). The epidemic of the 20th century: coronary heart disease. Am J Med.

[5] Cappelletti A, Latib A, Mazzavillani M, Magni V, Calori G, Colombo A (2012). Severity and prognostic localization of critical coronary artery stenoses: correlation with clinical control of major traditional risk factors. Coron Artery Dis.

[6] Sianos G, Morel MA, Kappetein AP, Morice MC, Colombo A, Dawkins K (2005). The SYNTAX Score: an angiographic tool for grading the complexity of coronary artery disease. EuroIntervention.

[7] Wilson PW (1994). Established risk factors and coronary artery disease: The Framingham Study. Am J Hypertens.

[8] Fischer M, Broeckel U, Holmer S, Baessler A, Hengstenberg C, Mayer B U (2005). Distinct heritable patterns of angiographic coronary artery disease in families with myocardial infarction. Circulation.

[9] Pencina MJ, Navar AM, Wojdyla D, Sanchez RJ, Khan I (2019). Quantifying importance of major risk factors for coronary heart disease. Circulation.

[10] Larifla L, Armand C, Velayoudom-Cephise FL, Weladji G, Michel CT, Blanchet-Deverly A (2014). Distribution of coronary artery disease severity and risk factors in Afro-Caribbeans. Arch Cardiovasc Dis.

[11] Neumann F-J, Sousa-Uva M, Ahlsson A, Alfonso F, Banning AP, Benedetto U (2019). 2018 ESC/EACTS Guidelines on myocardial revascularization. Eur Heart J.

[12] Pourhoseingholi MA, Vahedi M, Rahimzadeh M (2013). Sample size calculation in medical studies. Gastroenterol Hepatol Bed Bench.

[13] SYNTAX Score Calculator [Internet].

[14] American Diabetes Association (2018). 2. Classification and diagnosis of diabetes: standards of Medical Care in diabetes—2018. Diabetes Care [Internet].

[15] Williams B, Mancia G, Spiering W, Agabiti Rosei E, Azizi M (2018). 2018 ESC/ESH Guidelines for the management of arterial hypertension. Eur Heart J.

[16] Nayor M, Vasan RS (2016). Recent update to the US cholesterol treatment guidelines: a comparison with international guidelines. Circulation.

[17] Who.int (2008). Waist circumference and waist-hip ratio: report of a WHO expert consultation.

[18] Cappelletti A, Astore D, Godino C, Bellini B, Magni V, Mazzavillani M (2018). Relationship between Syntax Score and prognostic localization of coronary artery lesions with conventional risk factors, plasma profile markers, and carotid atherosclerosis (CAPP Study 2). Int J Cardiol.

[19] Hansson GK (2005). Inflammation, atherosclerosis, and coronary artery disease. N Engl J Med.

[20] Chiha J, Mitchell P, Gopinath B, Plant AJH, Kovoor P, Thiagalingam A (2015). Gender differences in the severity and extent of coronary artery disease. Int J Cardiol Heart Vasc.

[21] Tanaka T, Seto S, Yamamoto K, Kondo M, Otomo T (2013). An assessment of risk factors for the complexity of coronary artery disease using the SYNTAX score. Cardiovasc Interv Ther.

[22] El Kersh AM, Reda AA, El Hadad MG, El-Sharnouby KH (2018). Correlation between SYNTAX score and pattern of risk factors in patients referred for coronary angiography in Cardiology Department, Menoufia University. World J Cardiovasc Dis.

[23] Yadav M, Palmerini T, Caixeta A, Madhavan MV, Sanidas E, Kirtane AJ (2013). Prediction of coronary risk by SYNTAX and derived scores: SYNTAX. J Am Coll Cardiol.

[24] Cappelletti A, Astore D, Godino C, Bellini B, Magni V, Mazzavillani M (2018). Relationship between Syntax Score and prognostic localization of coronary artery lesions with conventional risk factors, plasma profile markers, and carotid atherosclerosis (CAPP Study 2). Int J Cardiol.

[25] Bots SH, Peters SA, Woodward M (2017). Sex differences in coronary heart disease and stroke mortality: a global assessment of the effect of ageing between 1980 and 2010. BMJ Glob Health.

[26] Málek F, Dvořák J, Skalníková V, Mates M, Kmoníček P, Vávrová Z (2015). Correlation of lipoprotein (a) with the extent of coronary artery disease in patients with established coronary atherosclerosis: gender differences. Eur J Prev Cardiol.

[27] Raal FJ, Alsheikh-Ali AA, Omar MI, Rashed W, Hamoui O, Kane A (2018). Cardiovascular risk factor burden in Africa and the Middle East across country income categories: a post hoc analysis of the cross-sectional Africa Middle East Cardiovascular Epidemiological (ACE) study. Arch Public Health.

[28] Veeranna V, Pradhan J, Niraj A, Fakhry H, Afonso L (2010). Traditional cardiovascular risk factors and severity of angiographic coronary artery disease in the elderly. Prev Cardiol.

[29] Bhattacharyya PJ, Vijapur S, Bhattacharyya AK (2016). A Study of cardiovascular risk factors correlation with the angiographic severity of coronary artery disease using Syntax score. IOSR Journal of Dental and Medical Sciences.

[30] Bruce SP, Acheampong F, Kretchy I (2015). Adherence to oral anti-diabetic drugs among patients attending a Ghanaian teaching hospital. Pharm Pract (Granada).

[31] Koliaki C, Sanidas E, Dalianis N, Panagiotakos D, Papadopoulos D, Votteas V (2011). Relationship between established cardiovascular risk factors and specific coronary angiographic findings in a large cohort of Greek catheterized patients. Angiology.

[32] Parsa AF, Jahanshahi B (2015). Is the relationship of body mass index to severity of coronary artery disease different from that of waist-to-hip ratio and severity of coronary artery disease? Paradoxical findings. Cardiovasc J Afr.

[33] Supariwala A, Uretsky S, Singh P (2011). A. Synergistic effect of coronary artery disease risk factors on long-term survival in patients with normal exercise SPECT studies. J Nucl Cardiol.

